# 439. Impact of viral load in HIV adult patients hospitalized with COVID-19

**DOI:** 10.1093/ofid/ofad500.509

**Published:** 2023-11-27

**Authors:** Alberto Romero Garcia, Chee Yao Lim, Jorge Gutierrez, Afsheen Afzal, Dwayvania Miller, Alexander La Fortune, Addi Feinstein, Vidya Menon

**Affiliations:** NYCHHC/Lincoln, New York, New York; NYCHHC/Lincoln, New York, New York; NYCHHC/Lincoln, New York, New York; Lincoln Medical Center, New York, New York; Lincoln Medical Center, New York, New York; NYCHHC/Lincoln, New York, New York; Lincoln Medical Center, New York, New York; Lincoln Medical Center, New York, New York

## Abstract

**Background:**

There is no difference in mortality in COVID-19 patients with and without HIV. The purpose of our study was to assess the outcomes of patients with HIV (PWH) and COVID-19 when stratified by their viral load (VL).

**Methods:**

In this retrospective single center study, data was collected from electronic medical records from March 2020 to December 2021. All adult patients admitted with a diagnosis of COVID-19 pneumonia were included. They were stratified according to their HIV status and subsequently PWH were stratified and analyzed according to their VL. We compared mortality rate, length of stay (LOS), incidence of septic shock, and severity of COVID-19 infection (by Quick COVID-19 Severity Index) using chi-square and we performed a multivariate analysis.

**Results:**

During this period a total of 1902 patients were admitted for COVID-19 infection and 78 patients were PWH. 32 patients had undetectable VL vs 46 with detectable VL. Baseline characteristics are described in table 1. PWH had a mortality rate of 15% (12 out of 77) compared with 27% (508 out of 1824) of the rest of population P=0.016. Among PWH, when stratified by their viral load, patients with detectable viral load had lower mortality incidence (4.3% vs 32.4% p=0.001), lower incidence of septic shock (5% vs 23%, p=0.015), and lower rates of severe and critical COVID-19 infection (52% vs 80.6%, p=0.011). Analysis by logistic regression was conducted to determine the impact of age and other comorbidities on mortality risk.. No association was found between VL status and incidence of mortality, septic shock, or severity of COVID-19. Length of stay and COVID-19 severity had no significant difference between these groups.

**Figure:**
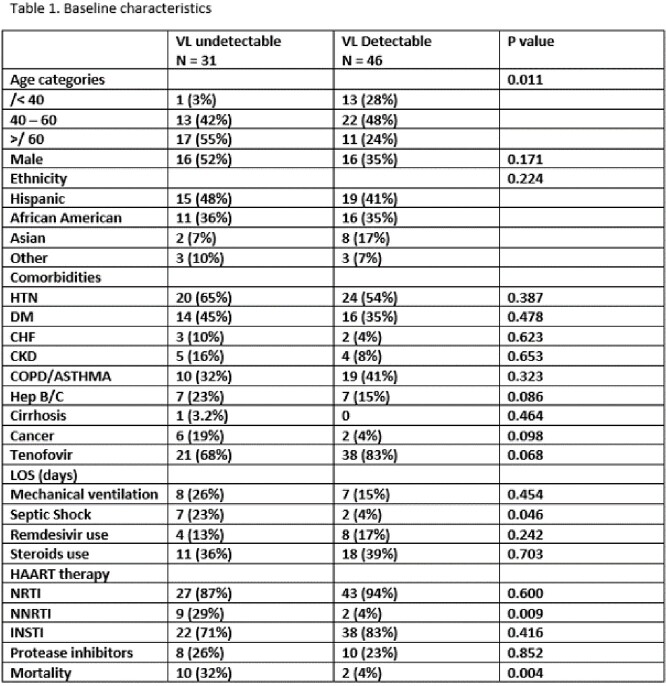

**Conclusion:**

It was thought that PWH with detectable viral load experienced less severe COVID-19 disease course due to the unsuppressed disease by the defective immunity and ART, as evidenced by observed lower rates of mortality and complications like septic shock. However, our study found no significant impact of VL in PWH on COVID-19 outcomes when accounting for age and comorbidities. These findings emphasize the need to consider the higher prevalence of comorbidities in PWH and other social determinants of health when examining the course of COVID-19 infection in an aging HIV population.

**Disclosures:**

**All Authors**: No reported disclosures

